# The impact of a community‐based music intervention on the health and well‐being of young people: A realist evaluation

**DOI:** 10.1111/hsc.12931

**Published:** 2019-12-25

**Authors:** Francesca Caló, Artur Steiner, Stephen Millar, Simon Teasdale

**Affiliations:** ^1^ Yunus Centre for Social Business and Health Glasgow Caledonian University Glasgow UK; ^2^ School of Music Cardiff University Cardiff UK

**Keywords:** community‐based music interventions, participation, realist evaluation, well‐being, young people

## Abstract

In recent years, music‐based interventions have been utilised as a tool for improving public health, reducing inequalities and promoting well‐being of young people. Although some researchers have begun to draw links between music‐related interventions and positive health outcomes, there is little understanding as to how such effects are produced. Realist evaluations—understanding what works, for whom and under what circumstances—are a particularly apt means by which we can open this ‘black box’. In this paper, we use a realist evaluation to assess a community‐based music initiative designed and implemented to support the well‐being of disadvantaged young people in Scotland. In order to gain perspectives on the range of contextual characteristics, mechanisms and outcomes, we collected quantitative and qualitative data in the form of pre‐ and post‐questionnaires, as well as conducting interviews with beneficiaries and stakeholders. Our findings show that the intervention achieved a positive impact on the self‐confidence, well‐being and engagement of disadvantaged young people. This impact was achieved via an approach personally tailored to the individual needs of the young people; and an organisational environment characterised by trust, whereby young people felt safe to express themselves.

**What is known about this topic**

- Well‐being of disadvantaged young people is an important issue in contemporary health policy.
- Community‐based initiatives have gained prominence as a means to positively influence individuals’ physical and psychological health.
- Existing research demonstrates that music interventions can lead to improvements in self‐efficacy
- Self‐esteem
- Well‐being and health of adolescents. However little is known about how these outcomes are triggered.

**What this paper adds**

- The contexts and mechanisms by which community‐based music interventions can lead to positive health and well‐being outcomes for disadvantaged young people;
- Pathways through which a flexible and bespoke music interventions can help to establish a space where disadvantaged young people feel protected
- Enhance their engagement with their peers and mentors and increase their self‐confidence;
- The importance of staff with relevant expertise and background in triggering the establishment of a self‐identification path which helps to build‐up trust between disadvantaged young people and the intervention participants

## 1 INTRODUCTION

In the UK, community‐based initiatives have gained prominence as a potential means to positively influence individuals’ physical and psychological health (Adebayo, Salerno, Francillon, & Williams, 2018; Brunton et al., 2017). Used as a tool for promoting social inclusion, arts and music have been associated with the enhancement of well‐being (Boyce, Bungay, Munn‐Giddings, & Wilson, 2018). As such, participation in creative arts has been recognised by health policies as one possible instrument for reducing health inequalities and improving public health (Atkinson & Robson, 2012).

Studies which link young people and music are diverse and heterogeneous, covering different areas of music, practice and discipline (McFerran, Garrido, & Saarikallio, 2016). Some studies have explored the effectiveness of music interventions in supporting adolescents with psychopathologies (Albornoz, 2011; Gold, Saarikallio, Crooke, & McFerran, 2017; Gold, Voracek, & Wigram, 2004; Gold, Wigram, & Voracek, 2007), whereas others have analysed how these interventions can improve social connectedness and self‐esteem among adolescents with mental health problems and behavioural problems (Hense & McFerran, 2017; McFerran et al., 2016; Porter et al., 2017). Researchers have also considered the role of community‐based initiatives promoting arts and music in addressing problems of disadvantaged young people (Harkins, Garnham, Campbell, & Tannahill, 2016; Parker, Marturano, O’Connor, & Meek, 2018), in youth custodial (Daykin, Viggiani, Moriarty, & Pilkington, 2017) and in community settings (Davies et al., 2013).

While studies show encouraging results in terms of the impact of community interventions on self‐efficacy, self‐esteem, well‐being and health of adolescents, the evidence linking young people's participation in the arts to increased engagement and well‐being is fragmented and inconclusive (Jindal‐Snape et al., 2018; Stickley et al., 2017). This suggests a need to strengthen the evidence base through the application of rigorous methodological approaches (Crooke & McFerran, 2014). Although quantitative studies including pilot surveys and randomised controlled trials have been conducted (Gold et al., 2017; Porter et al., 2017), questions remain about the utility of such approaches in evaluating the impact of (what are contextually dependent) music interventions on well‐being (Crooke & McFerran, 2014; DeNora & Ansdell, 2014). More importantly, and in relation to disadvantaged young people, existing studies do not answer ‘how’ music interventions impact aspects of well‐being and engagement and ‘what’ are their pathways. As such, the evidence regarding processes associated with the impact of community‐based music interventions remains unclear (Jindal‐Snape et al., 2018; McFerran et al., 2016).

Considering existing knowledge gaps, this study aims to assess the impact of a community‐based intervention on the well‐being and engagement of disadvantaged young people, identifying not only what outcomes were produced, but also how they were produced, and the significance of context. Through a realist evaluation approach (Pawson & Tilley, 1997), we present context‐mechanism‐outcome (CMO) configurations of a specific intervention conducted in four community settings. In our paper, first we turn attention to the methodology used in the realist evaluation, before analysing pathways of CMO configurations (findings) derived by our data analysis. In the discussion section, we explore whether, how and why community‐based music initiatives might have an impact on well‐being outcomes of disadvantaged young people, before identifying the implications for policy makers, researchers and practitioners.

## 2 METHODOLOGY AND METHODS

The methodology chosen to address the aims and objectives of this research emphasises the role of context and mechanisms in shaping outcomes. Realist evaluations are based on the identification of the outcome patterns, generative mechanisms and contextual conditions which help to assess not only what works, but also for whom, and in what circumstances (Pawson, 2006; Pawson & Manzano‐Santaella, 2012; Pawson & Tilley, 1997). This approach focuses on building and refining programme theories concerning complex casual mechanisms and exploring how these mechanisms interact with contextual and individual characteristics (Fletcher et al., 2016). The task of a realist evaluation is to explore the ‘black box’ of programmes (Salter & Kothari, 2014). In this paper we follow the recent ‘RAMESES’ (Realist And MEta‐narrative Evidence Syntheses: Evolving Standards) II guidelines and publication standards (Wong et al., 2017).^1^ We opted for a mixed‐methods research approach, which included the collation and analysis of qualitative interview and quantitative questionnaire data. Ethical approval was obtained from the University's Ethics Committee. Confidentiality and anonymity of all participants was maintained throughout the collection of all interviews and questionnaires, analysis and reporting. Pseudonyms of participants are used in all quotes.

### 2.1 Research setting

The focus of this study is on a community‐based intervention provided by a community organisation called Heavy Sound, based in Scotland. Through participatory music making, the organisation aims to engage disadvantaged young people in creative activities helping them to express their emotion. The specific community‐based intervention, COOL Music^2^ has received funding from the Scottish Government and the European Social Fund to run sixteen sessions of participatory music‐making in four different settings (one school, two community centres and one charity). Researchers at [*University and Department*] were funded to undertake an evaluation of the programme. To facilitate the development of the project, the research team maintained a close relationship during the project period with Heavy Sound managers, who kept them informed about aspects associated with the implementation of the intervention, and shared emerging findings. In this paper, we present findings deriving from 27 young people, aged 12–17 years old, from different deprived communities and with adverse life experiences.

### 2.2 Data collection

Quantitative and qualitative data were collected between April and September 2018. COOL Music interviewees were recruited directly by researchers at the beginning of the intervention. The research was explained to, and an information sheet shared with, potential study participants. Participation in the study was voluntary. Those who agreed to participate, completed pre‐ and post‐project questionnaires.

The pre‐ and post‐evaluation questionnaire was undertaken to assess changes in well‐being. The questionnaire used 8 out of 10 domains from the Good Childhood Index (Ress, Goswami, & Bradshaw, 2010) questionnaire: family, health, friendship, home, time use, school, appearance and future. The questionnaire also included the life satisfaction measures of personal well‐being as used by ONS National Wellbeing Programme. The questionnaire was tested and compared with a number of other tools measuring children's well‐being and proved to have a good internal consistency (Cronbach's alpha of 0.84), good reliability and validity (Rees, Goswami, & Bradshaw, 2010). In our sample, 23 young people out of 27 completed the pre‐test questionnaire and 18 out of 23 responded also to the post‐test questionnaire. Although relatively limited in its size, our sample reflected the nature of community‐based initiatives that tend to be small. Also, considering our realist evaluation approach, the sample was sufficient in identifying positive and negative outliers.

In line with realist evaluation, the qualitative component of the study included views of stakeholders because of their help to provide insights into the complexity of an initiative—identifying aspects that would not otherwise be revealed. As such in‐depth semi‐structured qualitative interviews were conducted. Different points of views were pursued to ensure the inclusion of a variety of perspectives. A total of 37 interviews involving young beneficiaries of the project (*n* = 23), Heavy Sound leaders and workers (*n* = 6), educators from other organisations, support/social workers of the young people and school teachers (*n* = 8) were conducted. These were recorded and transcribed (Saldaña, 2015). Table 1 summarises study participants and the type of collected data.

**Table 1:** Participants and data

Participants	Data collected
Young people	*Quantitative*: 23 pre‐test questionnaires, 18 post‐test questionnaires *Qualitative*: 23 interviews with beneficiaries of the intervention
Heavy Sound leaders and workers	*Qualitative*: Six interviews with COOL Music leaders and those delivering the intervention (note: all worked and were familiar with the young people as practitioners)
Educators, social workers and school teachers	*Qualitative*: Eight interviews conducted with four educators, three social workers and one teacher (note: all worked and were familiar with the young people as practitioners)

The interview guide consisted of a range of 17–22 open‐ended questions, depending on the stakeholder groups involved. The guide was divided into three domains of inquiry, following a realist evaluation approach, that aimed to (a) analyse generative mechanisms behind the intervention; (b) study the contextual variables embedded in the intervention and (c) explore the outcomes patterns.

### 2.3 Data analysis

Questionnaire data were analysed in a quantitative data analysis software. The researchers undertook a descriptive analysis of the Good Childhood Index domains, alongside a comparison with the mean results at a national level. In addition, an analysis of variation in data over time was conducted through the use of non‐parametrical analysis. No significant positive or negative changes were found, thus, an analysis of the outliers was undertaken. The identification of positive and negative outliers helped, through a realist approach, to explore why the intervention worked particularly well (or not) for specific participants (Prashanth, Marchal, Devadasan, Kegels, & Criel, 2014). This analysis is reported in the findings section to corroborate and triangulate the qualitative findings and the identification of the CMO configurations.

Interview data were analysed in qualitative data analysis software. Explanations for findings were generated in an abductive fashion ‘by moving backward and forward among empirical data, research literature, and emergent theory’ (Dey & Teasdale, 2013, p. 255). To facilitate this process, interviews were initially coded separately in terms of statements related to contexts, mechanisms and outcomes, following a typical thematic analysis process (Saldana, 2016). In the second round of coding, we employed ‘linked coding’ to establish whether it would be possible to generate CMO configurations directly from the narrative accounts of those interviewed (Jackson & Kolla, 2012).

## 3 FINDINGS

Our findings have been grouped into two key CMO configurations. The first grouping relates to the contextual characteristic of the community‐based intervention, the promotion of *a polyhedral and boutique approach*, which included staff with different expertise, background and musical preferences/skills. The second grouping relates to a key mechanism identified to engage disadvantaged young people: *feeling safe.*

**Figure 1:**
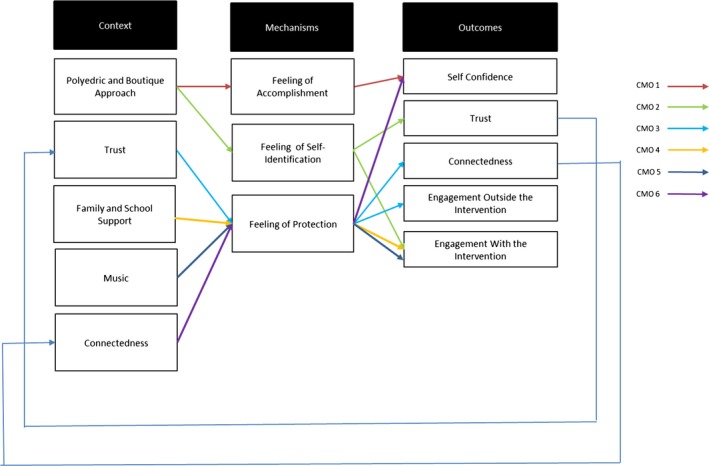
The theory programme

Figure 1 summarises our Programme Theory, developed from a synthesis of the six CMO statements which will be detailed below.

### 3.1 Polyhedral and boutique approach

**CMO Configuration 1**: Heavy Sound was identified as promoting a polyhedral and boutique approach (context). This characteristic triggered feelings of accomplishment (mechanism) among beneficiaries, which helped to increase their self‐confidence (outcome).

#### 3.1.1 Context

Support workers, educators and COOL Music staff recognised that Heavy Sound promoted a person‐centred intervention that included a synergic presence not only of different music expertise and styles, but also of personalities and approaches. Each of the young people required a tailored service, which implied ‘a boutique approach’ to their learning as highlighted by one of COOL Music worker:

Every single person requires a degree of a bespoke, boutique approach to their learning. Although there is group‐work, there is also a lot of one‐to‐one work in the sessions. I am able to completely adapt my approach to each person. And each person has required different levels of [attention]… maybe I have to repeat something, maybe I have to say things in a different way that they’ll understand. [COOL Music worker]

Some workers conducting the intervention were experts in specific musical styles, or in playing particular instruments. Others, including the organisation's founder, specialised in encouraging young people to use music to explore their own emotions and problems. This heterogeneity and synergy of roles was recognised by the support workers and educators as a distinct characteristic of the community‐based intervention.

#### 3.1.2 Mechanism

A degree of adaptability to accommodate needs of the young people and the promotion of individualised services were perceived as key factors to engender a sense of accomplishment. At the beginning of the intervention, each of the young people involved decided what they wanted to learn during the 16‐week course. Examples included music recording, song‐writing and learning how to play an instrument:

You can go off and do your own thing. You don’t just have to sit and learn something that you don’t want to learn. [Beneficiary]

Because the project was participant‐led, young people felt they were able to achieve the objectives they set for themselves, without being involved in something that was not of interest to them. Whether it was singing, spoken‐word, playing instruments, or utilising music technology, exploring their own development pathway made participants feel accomplished in achieving their goals and objectives:

Aye, I feel accomplished. I feel like I’ve done what I want to do. [Beneficiary]

#### 3.1.3 Outcomes

The sense of accomplishment derived from being able to achieve something tangible that they can read, listen to and improve, was identified as a pathway to increasing young people's self‐confidence, as highlighted by one of the school teachers:

For some of them it [the project] had helped find their sense of self‐worth, raised their confidence, and their successes and the way it’s planned out and they had so many successes as they went that raises confidence in themselves, and the young people who were like ‘I don’t want to be in a room where there’s a guitar’, to picking up a guitar. [School teacher]

For example Robert, who is autistic and struggled to express himself in front of his peers, was encouraged to use his rudimentary knowledge of the kazoo to contribute to the music project. Initially, Robert struggled to speak about the problems and challenges he faced in his daily life. However, he was able to overcome this by speaking into his kazoo. Heavy Sound practitioners used this as a stepping stone to draw him into larger discussions, eventually incorporating him into a group performance. This sense of accomplishment from being able to take part and interact with his peers increased Robert's confidence, enabling him to perform on a stage. The increased sense of confidence and related well‐being was also confirmed by his survey results. Robert presented the largest increase between the pre‐and post‐test well‐being scores. Timothy, another participant, formally struggled to leave his home on account of prolonged bullying. Following his participation in COOL Music, Timothy achieved enough confidence to obtain a placement in a local business and to self‐travel both to the project and at work.

**CMO Configuration 2**: The presence of managers with different expertise, background, musical taste—polyhedral approach—(context) triggered the establishment of a self‐identification path (mechanism) which helped to build‐up trust between beneficiaries and the intervention (outcome).

#### 3.1.4 Context

The different expertise of COOL Music team members promoted a synergic environment in which each of the workers had a ‘specific and different hat’ on. The organisation leader was, for example recognised as the best person to deal with the traumatic experiences of the young people, whereas another worker was identified as the person to play and write music with. That ‘specific hat’ was recognised by the young people who had the opportunity to choose if they wanted to talk about their problems or if they just wanted to play specific music, write songs or use sounds technology:

One of the kids had an extremely bad day—he did not want to sit with me, didn’t want to do music because he felt so rotten, but he just sat with [the organisation founder] and talked to him. And that was brilliant, because he was able to do that. That only happened once, as well. And then another week he would come and be with me and didn’t want to talk about his problems, he just wanted to do music. [COOL Music worker]

#### 3.1.5 Mechanism

The synergic approach of the COOL Music team helped to establish a self‐identity path, and this was observed as one of the distinct mechanisms of the intervention. Young people recognised common ground with adults and understood that they could learn and exchange knowledge with them. They identified with the workers of the organisation because of their musical taste, past life experience or specific attitude. This helped to create a sense of connection and identification:

[The young person] has taken a bit of a shine to [one of the workers] because she’s quite alternative. She’s not your bog standard run‐of‐the‐mill worker. […]She listens to the same music and for [the young person] not having that much of a friendship circle or friendship group, it’s nice to see that there are older people, there are workers that like the same things as her. [Support Worker]

#### 3.1.6 Outcomes

A feeling joint interests and identification developed a profound sense of connection. Participants believed they could trust COOL Music staff, who provided a safe space where they felt nurtured and understood:

That was a big issue for him that he didn’t trust anyone. I think he’s gained that element of, “actually no, I can trust people again and these people aren’t going to judge me from what’s happened before. They’re going to judge me for me and the person that I put over now”. [Support worker]

The passion and encouragement of COOL Music's founder, alongside the self‐identification with his life experiences, prompted participants to speak about their own life experiences. While at the start of the intervention some of the young people did not want to write songs with specific meanings, at the end, they began to disclose their own emotions and became engaged in deep reflections.

### 3.2 Feeling safe

**CMO Configuration 3**: The trust relationship developed between COOL music and beneficiaries (context) contributed to safe feelings in the young kids (mechanism) leading to better connectedness and engagement with the intervention and with their own communities (outcomes).

#### 3.2.1 Context

Establishment of trust between beneficiaries and the COOL Music team was not only an outcome derived from the feeling of identification described above, but became a contextual and fundamental characteristic of the intervention. The young people involved in the music sessions, although at different levels, had all experienced trauma in their lives. This trauma negatively affected their level of trust towards adults. Young people felt the need to continuously test if the adults they worked with were trustworthy and safe:

What a lot of these young people experienced trauma, they’ve experienced inconsistency, and they will test you as a character to find out if you’re a safe character and if you’re going to be somebody that can be trusted… [Organisation leader]

#### 3.2.2 Mechanism

A trustworthy relationship between COOL Music staff and the young people participating in the project contributed to developing feelings of safety and protection. An unconditional positive approach allowed young people to feel free to discover new musical styles, or to disclose traumatic parts of their life without fear of judgement:

With that nurture, with that support, with having a person‐centred approach that’s based on unconditional positive regard, no matter what they do, what they’ve done, it allows them to become safe and secure with us, and allow… for them to start making disclosures where they normally wouldn’t, maybe looking at certain types of writing styles. [Organisation leader]

#### 3.2.3 Outcomes

The recognition of being in a safe and protected space generated connectedness among beneficiaries. Through the intervention, some of young people who were bullied or experienced loneliness felt safe enough to get to know people and develop new friendships. These friendships continued outside the COOL Music space:

This project… definitely brought them all together, where a lot of them where maybe just living their wee single life playing their PlayStation and not coming out their house, but now the group as a whole have got a good bond together. They’re doing everything together. They’re meeting up after the project too and hanging about together, so it’s dragging them away from their house which is all good for their well‐being’. [Support Worker]

One of the project participants, Charlotte, highlighted that without the project she would have not met one of her friends and that they would not have been able to help each other with issues outside of the project. Other young people instead decided to reengage with their own communities, whether that meant going to school more often, taking up a placement in a local business, or participating in other projects.

**CMO Configuration 4**: The support of the family, friends and/or the school (context) contributed to promote feeling of protection (mechanism) which helped beneficiaries to engage more with the intervention (outcome).

#### 3.2.4 Context

Family, friends and school support were all identified as important contextual factors to encourage the young people to be part of the intervention. For example support workers identified that the presence of friends, or people that beneficiaries already knew from other projects and contexts, reduced young people's reluctance of joining a new and unknown space. The commitment of the school was also identified as a characteristic that could affect the engagement in COOL Music. For example John stressed that the encouragement from his ex‐music teacher (and now Deputy Head Teacher of the school) to take part in the intervention facilitated his participation in the programme:

I trust my Deputy Head—I’m quite close with my Deputy Head, if she thinks it’s something good for me to do, then I’ll just do it […] But this—I do really enjoy this. [Beneficiary]

#### 3.2.5 Mechanism

Receiving support from people that beneficiaries perceived as trustworthy helped the young people to feel safe in an unknown space, contributing to feelings and protection as set out above. Friends, teachers and educators therefore played an important role in turning a new and unfamiliar place into a ‘fearless zone’ where participants felt free to express their feelings:

For the kids that I’ve brought along, them knowing [Sarah] and that they already know someone here, so they’ve not been reluctant to come along because they don’t know anyone, and they know other kids actually because the other kids were involved at different meetings with another intervention. [Support worker]

#### 3.2.6 Outcomes

The feeling of protection deriving from the support offered by the ‘external trustworthy people’ incentivised the commitment and engagement of the young people:

They [young people] were interested in what each other was doing, some people would come in to support the other one when they were recording, if they didn’t feel confident enough to sing, to encourage them to do that. And that kind peer support is really powerful and it wouldn’t work if it was a teacher saying, “can you give it a try.” If their friends are actually saying “oh, it’s really good,” “you’ve got to do this” you get a better result. [Support worker]

Without additional support, young people struggled to be engaged with the intervention. For example Johanna found it challenging to build positive relationships with the other participants in her group. This affected her poor engagement in the intervention. Although we did not have the opportunity to interview her (she did not agree to be interviewed), her well‐being scores decreased between the pre‐and post‐test. She was a negative outlier in friendship, school and appearance categories of the Good Childhood Index domains.

**CMO Configuration 5**: Music (context) helped to establish a space where beneficiaries felt protected (mechanism) enhancing their engagement with the intervention (outcome).

#### 3.2.7 Context

Music was perceived as a space where participants felt free to express their own feelings without having to articulate their problems. One of the educators highlighted that music was just a bi‐product of the intervention and it was the hook to engage disadvantaged young people in a space where they could express themselves:

Music is a massive thing for young people nowadays to give them an outlet to be able to express themselves in a different way. [Educator]

COOL Music beneficiaries confirmed that music was the main reason for their initial interest and engagement with the intervention:

I love doing it. If you really appreciate music like I do, if anything like this came up, you’d be keen to do it. [Beneficiary]

#### 3.2.8 Mechanism

The young people were free to use metaphor and stories in their lyrics, without people forcing them to explore and explain what they really meant. Music became a tool used to manifest feelings and identity. Writing songs and/or composing lyrics was identified as a space where young people feel free to explore their lives, emotions and experiences:

It’s about understanding and accepting and exploring a little bit more about what their care identity means and some of the experiences that have happened to them, and I guess just using that in a really expressive way to better manage emotions and all those interpersonal skills that go on. [Educator]

#### 3.2.9 Outcomes

The possibility of expressing own feelings through music not only increased during the intervention, it also provided an instrument to communicate with carers about what participants were thinking and the lives they were living:

It was quite emotional actually listening to [Tom’s song] because you don’t hear them expressing their emotions on a day to day basis at work but coming here I’ve seen them and thought “Oh, that’s what you think’?” [Support worker]

**CMO Configuration 6:** Connecting with more people (context), reinforced the feeling of protection (mechanism), helping beneficiaries to increase their self‐confidence (outcome).

#### 3.2.10 Context

Throughout the project, each of the participants affirmed that they had the possibility to talk and meet with more people, engaging with both the group and the COOL Music team. Connectedness, then, from being an outcome explored in CMO Configuration 3 became a contextual characteristic:

If you're someone who wouldn't really have many friends or is getting bullied, joining this club^3^ would get you socialised and you'd probably make friends. And you'd just be able to cope with more stuff that happens. [Beneficiary]

#### 3.2.11 Mechanism

Connecting with more people, and learning how to engage with people that they did not know before, reinforced beneficiaries’ feelings of protection. Through informal conversations, the young people started to engage with COOL Music workers, first, before opening up to the entire group.

Compared to the first day, I wasn’t talking. The second day I started talking a wee bit. It was only to [two people COOL Music workers]. Third week started talking a wee bit more and now I think I’ve started talking quite a bit. [Beneficiary]

#### 3.2.12 Outcomes

Most of the project beneficiaries presented evidence that the possibility of meeting new people boosted their self‐confidence and increased their enthusiasm of being part of COOL Music:

I’ve met new people, so it’s boosted my confidence a wee bit. I made some new pals. [Beneficiary]

## 4 DISCUSSION

The aim of the study was to assess the impact of a community‐based music intervention on the well‐being and engagement of disadvantaged young people, identifying not only what outcomes were produced by the intervention, but also how they were produced, and the significance of context*.*

Based on the patterns derived from analysis of the interviews the results of the COOL Music project add weight to the existing literature on the positive effects of community‐based music interventions in increasing self‐confidence, well‐being and engagement of young people (Adebayo et al., 2018; Brunton et al., 2017; Daykin, Moriarty, De Viggiani, & Pilkington, 2011; Levy, Robb, & Jindal‐Snape, 2014; McFerran et al., 2016).

Our paper presents empirical data deriving from COOL Music—an intervention that developed a tailored polyhedral approach, which requires a high level of flexibility based upon specific needs of the young people. A community‐based organisation, which focuses on a small number of people, can give specific consideration and flexibility to its beneficiaries (Baker, Bull, & Taylor, 2018). The polyhedral approach of the music practitioners encouraged a path of self‐identification, which confirms the importance of music practitioners in establishing a trustworthy relationship with the consequent reinforcement of the engagement in the intervention (Harkins et al., 2016; Parker et al., 2018). This seems particularly important in the case of young people who have suffered traumatic experiences (McFerran, Roberts, & O Grady, 2010).

Second, *feeling safe* was the key mechanism to trigger most of the observed outcomes. COOL Music was considered as a free and safe space without judgemental attachment, operating outside formal and rigid structures. *Feeling safe* was triggered by four different contextual characteristics (i.e. trust, family and school support, music and connectedness) that together helped to establish a space where disadvantaged young people could feel nurtured. While some of these contextual characteristics, such as music, have been identified by the literature as possible sources of happiness and enjoyment (see, e.g. Harkins et al., 2016), our paper reveals more complex pathways (as also suggested in the literature by McFerran et al., 2016), which identify the space of protection as the main mechanism to well‐being and engagement of young people.

Finally, two of the contextual characteristics that trigger the *feeling safe mechanism*—trust and connectedness—were also identified as two of the main outcomes. Trust was achieved through the self‐identification mechanism, triggered by the polyhedral approach, and the important role of music practitioners. Due to their specific training, experience and professional skills, music practitioners were fundamental in promoting the feeling safe mechanism (Crooke & McFerran, 2014; Millar, Steiner, Caló, & Teasdale, 2019). Connectedness was promoted by the feeling of protection, described above and triggered by the establishment of trust, which became a contextual characteristic of the intervention self. This shows that community‐based initiatives often develop complex pathways which reinforce and reengage the achievement of well‐being and engagement through a virtuous cycle.

### 4.1 Strength and limitations

Our study consisted of only one community‐based music intervention and this makes generalisation to other settings challenging. The extent to which the results could be isolated and attributed to COOL Music is also limited. Although we have tried to investigate all pathways, other potential justifications explaining the efficiency of the programme could possibly be identified. For example participation in other interventions in the community could have impacted upon the activation of specific mechanisms. Also, not all the beneficiaries agreed to participate in the study and not all of the study participants completed post‐project questionnaires. For example beneficiaries who were afterwards identified as negative outliers declined our invitation to be interviewed. Possibly, their participation could add to our findings confirming (or not) some the identified CMO which might help to understand why, in some cases, the intervention did not work. Finally, it is useful to reflect on using a mixed‐method research approach when dealing with young people from deprived communities and those with adverse life experiences. As evidenced in other studies (e.g. Crooke & McFerran, 2014), collecting quantitative data through the use of longitudinal questionnaires can be challenging; while some study participants might not want to complete post‐project questionnaires, there is a risk that the young people might not interpret the questions consistently. Quantitative methods may also not be appropriate when working with a small heterogenous sample, as any differences between groups may not prove significant. Combining both quantitative and qualitative approaches through a realist lens helped us to minimise these limitations, helping reveal of what worked, for whom and in what circumstances. However, it should be noted that our findings are skewed towards ‘what works’ since we were unable to collect follow‐up data from those (such as Joanna) who benefitted least from COOL Music.

## 5 IMPLICATIONS FOR RESEARCH AND/OR PRACTICE

Our findings have implications for both practitioners and policy makers. Further studies exploring the impact of these initiatives should be promoted by policy makers to understand if these interventions, compared with more homogeneous approaches, provide better and/or different outcomes and lower costs. Practitioners should focus upon maintaining a boutique approach in their intervention, while recognising that a safe space has to be at the heart of any intervention serving disadvantaged young people.

## 6 CONCLUSION

In our paper we have shown that community‐based music interventions can have an impact on the self‐confidence well‐being and engagement of some disadvantaged young people, thanks to the establishment of a polyhedral approach and the development of a ‘safe feeling’, triggered by the achievement of trust and connectedness, and reinforced by the music space. Bespoke approaches that are closer to the beneficiaries seem more likely to positively impact upon the engagement and well‐being of disadvantaged young people. The pathways identified should be tested in future studies, to better understand how such interventions are context‐dependent. Our programme theory provides a platform for future studies and is worth further empirical attention. Testing the results in community‐based music interventions with different contextual characteristics and in different settings will enable exploration of whether the pathways change or not, while collecting longer term data will enable to identify if the outcomes are sustained.

## ENDNOTES

1 RAMESES II provides guidance on quality assurance and uniform reporting for improving quality and consistency of realist evaluation studies (Wong et al., 2017).

2 Acronym of ‘Community Orientated and Opportunity Learning Music’ project.

3 Although COOL Music was not a club *per* se, some of the participants described it that way, which fits with how it became a social intervention as much as a musical one.
